# A YOLOv12-based approach for automatic detection of cephalometric landmarks on 2D lateral skull X-ray images

**DOI:** 10.1038/s41598-026-43250-z

**Published:** 2026-03-10

**Authors:** Parth Dhananjay Akre, Yash Ganesh Ghavghave, Utkarsha Pacharaney

**Affiliations:** 1 Department of Artificial Intelligence and Data Science, Faculty of Engineering and Technology, Datta Meghe Institute of Higher Education and Research (Deemed to Be University), Wardha, Maharashtra 442001 India; 2 Department of Artificial Intelligence and Machine Learning, Faculty of Engineering and Technology, Datta Meghe Institute of Higher Education and Research (Deemed to Be University), Wardha, Maharashtra 442001 India

**Keywords:** Cephalometric analysis, Deep learning, Medical imaging, YOLOv12, Computer vision, Orthodontics, Anatomy, Computational biology and bioinformatics, Health care, Mathematics and computing, Medical research

## Abstract

Cephalometric analysis is the quantitative evaluation of skeletal and soft-tissue relationships on lateral skull radiographs; it underlies diagnosis, treatment planning, and growth assessment in orthodontics. The analysis hinges on cephalometric landmarks which are anatomical reference points whose 2-D coordinates are used to derive angles, distances, and ratios that guide clinical decisions. Manual identification of these landmarks is time-consuming where each image can take from 10 to 15 min and is subject to inter- and intra-examiner variability that can exceed 2 mm, propagating error into subsequent measurements. In recent years, artificial intelligence methods have advanced rapidly and are now widely adopted in medical imaging. This paper proposes an automatic landmark-detection pipeline built on YOLOv12, the newest iteration of the You-Only-Look-Once family. Trained and evaluated on a publicly available cephalometric dataset, the YOLOv12 model successfully localized 53.47% of landmarks within 1 mm and 80.57% within 2 mm.

## Introduction

Cephalometric analysis represents a cornerstone of orthodontic diagnosis and treatment planning, providing clinicians with essential quantitative measurements derived from standardized lateral skull radiographs. This analytical technique, first introduced by Broadbent in 1931, enables the systematic evaluation of craniofacial morphology through the precise identification and measurement of anatomical landmarks on two-dimensional X-ray images^1^. The accuracy of cephalometric analysis fundamentally depends on the precise localization of these landmarks, which serve as reference points for calculating angular and linear measurements that guide clinical decision-making in orthodontics, oral surgery, and maxillofacial treatment planning^2^.

Traditional cephalometric landmark identification has long relied on manual annotation by trained clinicians, a process that is inherently time-consuming, subjective, and prone to inter-observer and intra-observer variability^3^. This variability becomes particularly problematic when considering that modern orthodontic treatment planning requires sub-millimeter precision to achieve optimal therapeutic outcomes^4^. Furthermore, the increasing volume of radiographic examinations in clinical practice has created a growing demand for more efficient and standardized approaches to cephalometric analysis^5^.

Artificial intelligence (AI) represents a broad field of computer science focused on creating systems capable of performing tasks that typically require human intelligence, including pattern recognition, decision-making, and problem-solving^6^. Within this domain, machine learning (ML) constitutes a subset of AI that enables computers to learn and improve performance on specific tasks through experience and data, without being explicitly programmed for every possible scenario^7^. Deep learning, a specialized branch of machine learning, utilizes artificial neural networks with multiple layers to automatically learn hierarchical representations of data, making it particularly effective for complex pattern recognition tasks^4^. In medical imaging applications, convolutional neural networks (CNNs) have emerged as the dominant deep learning architecture due to their ability to automatically extract spatial features from images through learned filters and pooling operations^4,6^. The hierarchical feature extraction capability of CNNs makes them exceptionally well-suited for analyzing X-ray images, where they can identify low-level features such as edges and textures in early layers, while progressively learning more complex anatomical structures and spatial relationships in deeper layers, ultimately enabling precise localization of cephalometric landmarks within the complex radiographic anatomy^2^.

The emergence of artificial intelligence and computer vision technologies has opened new avenues for automating cephalometric landmark detection, promising to address the limitations of manual approaches while improving both efficiency and consistency^8^. Over the past two decades, researchers have explored various computational methods ranging from traditional image processing techniques to sophisticated machine learning algorithms^9^. Early automated systems primarily relied on template matching, edge detection, and statistical shape models, achieving moderate success but often struggling with image quality variations, anatomical anomalies, and the complex geometric relationships inherent in craniofacial structures^10^.

The advent of deep learning has revolutionized medical image analysis, with convolutional neural networks demonstrating unprecedented performance in various diagnostic imaging tasks^11^. In the context of cephalometric analysis, deep learning approaches have shown remarkable progress in landmark detection accuracy, with several studies reporting performance levels approaching or even surpassing human expert annotations. These advances have been facilitated by the development of large annotated datasets, improved computational resources, and increasingly sophisticated network architectures specifically designed for medical image analysis^12^.

Among the various deep learning frameworks, the You Only Look Once (YOLO) series has emerged as a particularly promising approach for object detection tasks in medical imaging. The YOLO architecture’s ability to perform real-time object detection while maintaining high accuracy makes it especially suitable for clinical applications where processing speed and reliability are paramount. The evolution from YOLOv1 through subsequent iterations has consistently improved detection performance, with each version incorporating architectural innovations that enhance both speed and accuracy^13^. The recent introduction of YOLOv12 represents the latest advancement in this series, featuring enhanced feature extraction capabilities, improved multi-scale detection, and optimized training strategies that are particularly relevant for medical image analysis applications^14^.

Recent advancements in YOLOv12 architecture have introduced several key improvements that make it particularly well-suited for cephalometric landmark detection tasks. The enhanced feature pyramid network and improved anchor-free detection mechanism allow for better handling of small anatomical structures and overlapping landmarks commonly encountered in lateral cephalograms. Additionally, the incorporation of advanced data augmentation strategies and improved loss functions in YOLOv12 training protocols has potential in addressing the challenge of limited annotated medical datasets, a common constraint in cephalometric research.

The clinical validation of YOLOv12-based systems for cephalometric analysis requires rigorous evaluation against established gold standards, typically involving expert manual annotations from experienced orthodontists. Current studies have reported promising results, with some implementations achieving landmark detection accuracies within clinically acceptable ranges of 1-2 mm for most anatomical points. However, significant variations in performance across different landmark types and patient populations highlight the need for continued research and refinement of these approaches to achieve consistent clinical-grade performance across diverse radiographic conditions and anatomical presentations.

The article is organized into several significant sections. It begins with an Abstract that outlines the purpose, methodology, and outcomes of the proposed YOLOv12-based cephalometric analysis framework. The Introduction provides background on cephalometric analysis, its role in orthodontic diagnosis and treatment planning, and the motivation for applying deep learning models to this domain. The Literature Review surveys earlier methods for cephalometric landmark detection, emphasizing both traditional image analysis techniques and recent CNN and YOLO-based approaches (Table 1). The Dataset Used section describes the cephalometric radiograph datasets employed, along with preprocessing and annotation strategies to ensure consistency and reliability. The Methodology details the YOLOv12 architecture, explaining its backbone, neck, and head components, and the workflow followed for training, inference, and evaluation. The Results section presents experimental findings with visual landmark detection outputs and performance metrics such as precision, recall, mAP, and localization accuracy. The Discussion highlights clinical applicability, comparing the proposed model with manual tracing and conventional diagnostic approaches, while emphasizing improvements in efficiency and reliability. The paper concludes with the Conclusion section, which summarizes contributions and clinical significance, and a Future Scope section that suggests directions for expanding datasets, improving robustness, and enabling real-time deployment in orthodontic practice. Finally, supporting works are compiled in the References section.

### Literature review

Recent literature indicates that AI-driven cephalometric tools can achieve high accuracy and efficiency, though outcomes depend on algorithm and context. Subramanian et al. (2022) report that modern AI algorithms substantially improve landmark-identification efficiency, accuracy, and reliability^15^. Likewise, Kiełczykowski et al.^10^ found that most AI approaches yield relatively high landmark‐detection accuracy and can expedite radiographic analysis, potentially aiding less experienced clinicians and shortening exam time. Hendrickx et al.^7^ confirmed these benefits in a systematic review: automated 2D landmarking errors averaged ~ 1.4 mm (below clinical thresholds) with processing under one minute, although they noted significant bias and heterogeneity in many studies. Kazimierczak et al.^6^ compared three commercial AI programs and observed very high agreement (ICC > 0.9) and perfect repeatability for most measures, but also found discrepancies in certain angular parameters, underscoring the need for standardization and careful clinician review^11^. Saifeldin et al.^3^ similarly found that fully automated AI tracing produced reliable and accurate measurements comparable to manual analysis, but cautioned that such technological advances cannot replace the orthodontist’s diagnostic judgment. Finally, Alessandri-Bonetti et al.^12^ noted that while experienced clinicians still achieved greater precision than AI, AI markedly reduced analysis time and error overall, confirming its promise but emphasizing the need for further validation and expert oversight. Overall, these studies converge on key themes: AI in cephalometry offers significant clinical potential (high accuracy and time savings), yet all underscore methodological limitations (e.g. small samples, algorithmic variability, bias) and the continued necessity of expert oversight when interpreting AI-generated results.

**Table 1:** Literature review.

Sr.No	Article Title, Author, and Publication Year	Study focus, design, goals, methodology, and sample size	The study’s findings and conclusions	The Scholar’s Remarks
1	“Cephalometric Analysis in Orthodontics Using Artificial Intelligence–A Comprehensive Review–Subramanian et al.^15^“	Focus: Applications of AI for cephalometric analysis in orthodontics. Design: Narrative review. Method: Electronic literature search (1980–2021); initially ~ 8420 records, with 64 publications	Findings: Rapidly evolving AI algorithms have markedly improved efficiency, accuracy and reliability of automated landmark identification in cephalometry. Conclusions: AI is a time-saving, reliable aid for routine cephalometric tracing and large-scale analysis, enhancing diagnostic workflows	Authors emphasize the review’s aim “to assist clinicians and researchers in comprehending various features of this study area”, highlighting AI’s potential benefits and the need for clinician familiarity with AI methods
2	“Application of Artificial Intelligence (AI) in a Cephalometric Analysis: A Narrative Review–Kiełczykowski et al.^10^“	Focus: Effectiveness of AI in orthodontic 2D cephalometric diagnostics. Design: Narrative literature review. Method: Searched PubMed, Medline, Scopus, and Dentistry & Oral Sci. Source (2009–2023); 23 studies included	Findings: Most AI algorithms achieved relatively high accuracy in localizing cephalometric landmarks. Conclusions: AI is a promising tool to facilitate landmark identification, support less-experienced clinicians, and shorten diagnosis time	Authors note that AI’s reliability “differs depending on the operator’s clinical training and experience, the number and quality of radiographs, [and] the type of algorithm”, and they predict continued AI development in orthodontics
3	“Reliability of Artificial Intelligence-Assisted Cephalometric Analysis–A Pilot Study–Alessandri-Bonetti et al.^12^“	Focus: Reliability of fully automated AI vs. manual computerized cephalometric analysis. Design: Retrospective pilot study. Method: 13 lateral cephalograms were analyzed by a fully AI-driven software (Novarad) and separately by a blinded operator using computerized digital software. Five cephalometric parameters were compared. Reliability (intra/inter-operator) assessed by Dahlberg’s formula and Bland–Altman analysis	Findings: No significant intra- or inter-method differences were detected. Fully automated AI-assisted analysis produced cephalometric measurements that were statistically reliable and accurate. Conclusions: Even with a small sample, AI-derived cephalometric values closely matched manual measures. Authors note that AI “were reliable and accurate,” although they observed non-significant trends of higher error in some measures	Authors conclude that “despite the small sample, the cephalometric measurements of a fully automated AI-assisted … software were reliable and accurate,” but they stress that such technology cannot substitute the clinician’s role in ensuring correct diagnosis
4	“Can AI-Driven Cephalometric Analysis Replace Manual Tracing? A Systematic Review and Meta-Analysis–Hendrickx et al.^7^.”	Focus: Accuracy and efficiency of AI-driven landmark detection versus manual tracing (2D lateral cephalograms and 3D CBCT). Design: Systematic review and meta-analysis. Method: Searched PubMed, Web of Science, Embase (to Jan 2024); 34 studies included (27 for 2D, 7 for 3D	Findings: Meta-analysis of 2D AI landmarking showed mean error ≈1.39 mm (95% CI 0.85–1.92 mm), below the 2 mm clinical threshold; 3D AI errors ranged 1.0–5.8 mm (high heterogeneity). Both 2D and 3D AI methods were very time-efficient (< 1 min per analysis). Many included studies had high risk of selection/reference bias. Conclusions: AI-driven landmark detection shows promising accuracy and speed, but existing systems require improved generalizability and robustness	Authors caution that most studies exhibited high bias and that AI performance, while promising, “could benefit from further improvement” in generalizability and robustness
5	“Comparison of Three Commercial AI-Driven Cephalometric Analysis Tools in Orthodontics–Kazimierczak et al.^11^.”	Focus: Compare CephX, WebCeph, and AudaxCeph (commercial AI tools) on orthodontic ceph metrics. Design: Retrospective study. Method: 124 lateral cephalograms from one orthodontic center. Automated analyses by each AI program using standard Downs, Ricketts and Steiner parameters; 50 cases re-analyzed to test repeatability. Agreement assessed by ICC and Friedman test	Findings: High agreement between AI systems for most parameters (ICC₃ > 0.9). Significant discrepancies appeared in angle convexity and occlusal plane measurements, indicating methodological differences. Each AI tool showed perfect intrasoftware repeatability (ICC≈1.0). Conclusions: AI-driven cephalometric tools can provide reliable, efficient assessments, but observed inconsistencies underscore the need for standardization and clinician review of automated results	Authors emphasize that discrepancies and variability in certain measurements “underscore the need for standardization across AI platforms and the critical evaluation of automated results by clinicians,” especially for parameters with strong treatment impact
6	“Discrepancies in Cephalometric Analysis Results between Orthodontists, Radiologists and Artificial Intelligence: A Systematic ReviewSmołka et al.^5^“	Focus: Compare cephalometric measurements from different specialists (orthodontists vs radiologists) and AI systems. Design: Systematic review. Method: Database search (PubMed, Scopus, WoS) for studies on cephalometric analysis discrepancies (to Jan 2024); 263 records screened, 17 studies included	Findings: Measurement accuracy strongly depended on operator expertise: experienced orthodontists/radiologists achieved greater precision than novices or non-specialists. Computer-assisted programs streamlined the workflow, reduced human error, and improved precision. AI-based analyses also showed high precision and markedly reduced analysis time. Conclusions: AI-assisted cephalometry can yield highly accurate, efficient results, but current systems still need enhancement. The review indicates further research is needed to optimize the integration of AI and software tools into orthodontic and orthognathic planning	Authors highlight that AI applications demonstrated “high precision and a substantial reduction in working time,” but also note that additional improvements and validation are required to fully harness technology in clinical practice

### Dataset used

This study employed two publicly available cephalometric datasets. The first dataset, obtained from the ISBI 2015 Challenge, consists of 400 lateral skull X-ray images with a resolution of 1935 × 2400 pixels^16^. Each image is annotated with 19 cephalometric landmarks, with two sets of annotations provided—one by a senior expert and another by a junior expert. To ensure higher reliability, only the senior expert annotations were used.

The second dataset comprises 102 cephalometric images of resolution 2089 × 1937 pixels, collected from an online repository^17^. Similar to the ISBI dataset, each image is annotated with 19 cephalometric landmarks, independently marked by two senior doctors. For consistency, the annotations from Doctor 1 were adopted in this work.

The annotated landmarks used in this study are Anterior nasal spine, Articulare, Gnathion, Gonion, Incision inferius, Incision superius, Lower lip, Menton, Nasion, Orbitale, Pogonion, Porion, Posterior nasal spine, Sella, Soft tissue pogonion, Subnasale, Subspinale, Supramentale, and Upper lip.

**Fig. 1:**
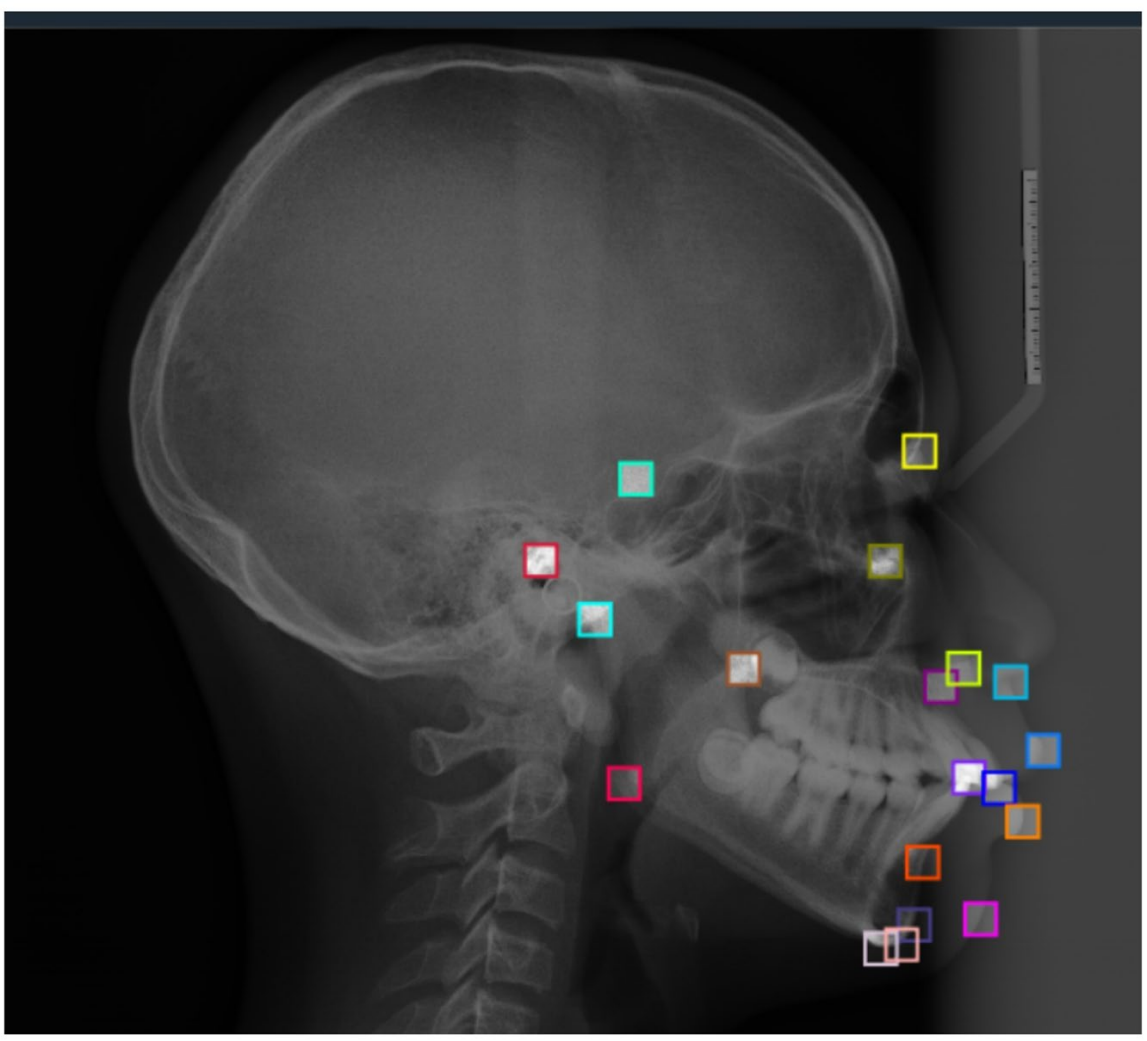
Lateral cephalogram with 19 bounding boxes representing the 19 cephalometric landmarks.

The two datasets were merged to create a unified collection of annotated cephalometric images. Since the original images were in BMP format, they were converted to PNG format to reduce storage size and enable faster training. Each image was paired with a text file containing the (x, y) coordinates of its 19 landmarks. To adapt these annotations for YOLOv12, a custom preprocessing script was developed to transform each landmark coordinate into a 58 × 58 pixel bounding box, where the landmark point was treated as the bounding box center (Fig. 1).

The final dataset was managed and prepared using Roboflow. Although the merged datasets initially contained 502 images, Roboflow’s similarity detection algorithm removed 3 duplicates, yielding a total of 499 images with bounding box annotations. A new dataset version was then created in Roboflow using data augmentation techniques. The augmentation settings included:

- Rotation: –3° to + 3°
- Shear: ± 2° (horizontal and vertical)
- Brightness adjustment: –10% to + 10%
- Exposure adjustment: –8% to + 8%
- Noise: up to 0.1% of pixels

The dataset initially comprised 499 images, was split into 218 training, 187 validation, and 94 test samples. To increase the variability of the training distribution, a 3 × augmentation procedure was applied exclusively to the training subset, expanding it to 654 images while preserving the original validation and test partitions. The augmented dataset therefore consisted of 935 images in total. The final dataset had 70% training, 20% validation, and 10% testing images.

## Methodology

**Fig. 2:**
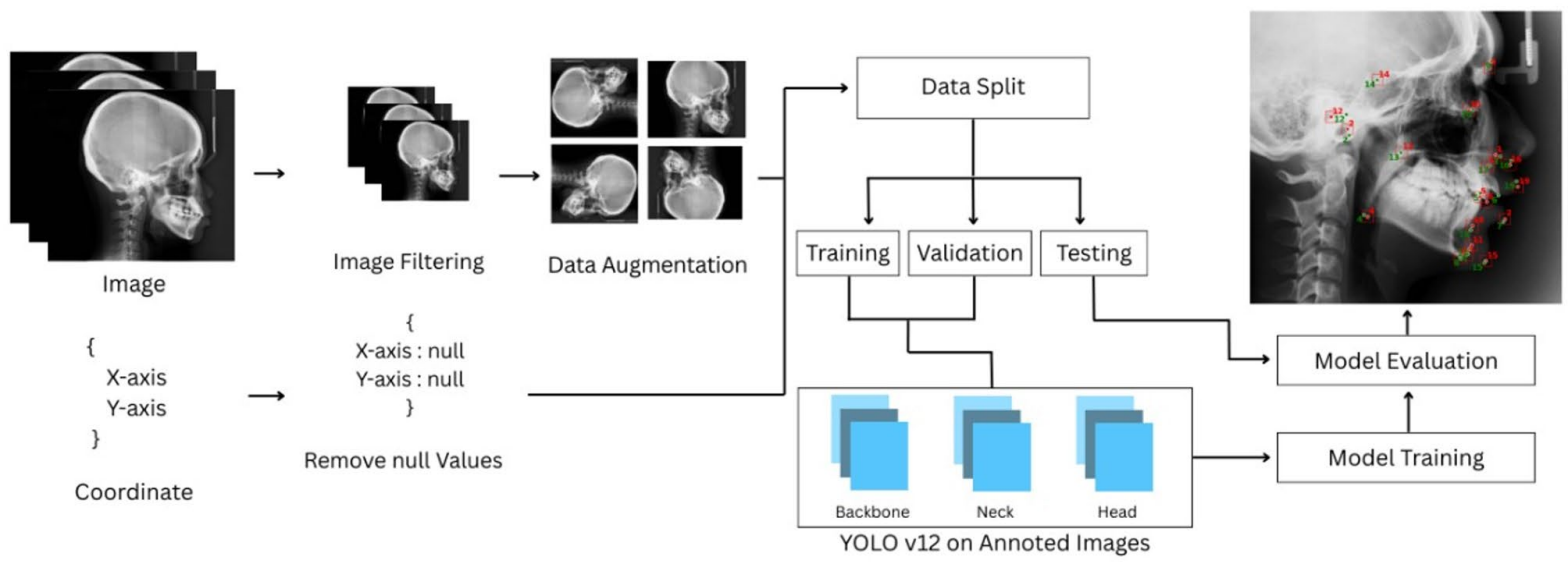
Cephalometric landmarks detection methodology framework.

The study’s methodology is a systematic pipeline with four primary steps: real-time cephalometric landmark detection, performance evaluation, YOLOv12 model training, and dataset preparation. Figure 2 depicts the entire procedure generally.

The proposed detection method relies on the YOLOv12 framework, which is an object recognition architecture recognized for its speed and accuracy. The single-stage detection process of the YOLOv12 model has three main parts:

### Backbone

The core of YOLOv12 gives the basic representations needed for detection tasks. It changes incoming picture data into multi-scale feature maps. Carefully positioned residual connections are combined with deeper convolutional layers in the Residual Efficient Layer Aggregation Network (R-ELAN)^14^. The backbone is made up of this. This technique increases the model’s ability to acquire minute details of objects of various shapes and sizes by overcoming gradient bottlenecks by improving feature reuse.

$$Fout = \sum\limits_{i = 1}^{n} {W_{i} * Fin + bi}$$

### Neck

In YOLOv12, the neck accumulates and refines multi-scale traits by acting as a conduit between the head and backbone. One of its main contributions is an area attention strategy that improves the model’s focus on important regions in crowded situations^13^. FlashAttention speeds up this technique.

### Head

Bounding box areas and detection scores are produced by the head of YOLOv12, which converts the improved feature maps generated by the neck through final predictions. The two main developments consist of tailored loss functions that optimize the trade between localization and classification tasks together with simplified approaches to multi-scale detection. A standard YOLO loss function receives enhancement through specific attention terms and confidence expressions ^13,14^.

*ℒ* = λcoord ∑(x^^^– x) + (y^^^– y) λobj ∑(c^^^– c) + …

Projected bounding box coordinates and confidence predictions are presented as x^ and y^ together with C^ in the output. The enhancements made to YOLOv12 lead to better real-time application performance.

### Model configuration

Training was performed using the YOLOv12 large variant (YOLOv12-l). The network complexity and compute footprint reported in the training log were: ≈26.46 million parameters and ~ 83 GFLOPs (pre-fusion: 895 layers; fused summary: 674 layers, 26,408,201 parameters, 82.1 GFLOPs). Input images were prepared at an operational size of imgsz = 640 with rectangular training enabled (rect = True) to preserve aspect ratios during batching. During the training run no additional online augmentation was enabled in the trainer (augment = False), since augmentation was performed earlier when preparing the dataset.

### Training procedure and hyperparameters

The model was trained for 50 epochs with a batch size of 12. Other training arguments passed to the Ultralytics training script were: lr0 = 0.005 (user-specified), lrf = 0.2, save_period = 10 (checkpoints saved every 10 epochs), project = ‘runs/cephalo_l’, and device = ‘0’. The Ultralytics trainer automatically selected an optimizer and adjusted the effective learning rate and momentum; the logs record that the automatic selection chose AdamW with an effective initial learning rate lr ≈ 4.35 × 10⁻^4^ and momentum = 0.9. The optimizer used three parameter groups with differing weight-decay settings (example groups: weight(decay = 0.0), weight(decay = 0.00046875), bias(decay = 0.0)), as reported by the training engine. Automatic Mixed Precision (AMP) checks were enabled and used during training to reduce memory use and accelerate mixed-precision computation.

### Hardware and runtime

Training was executed on a single GPU (reported device: Tesla T4, 15,095 MiB available). The complete 50-epoch run completed in ~ 1.280 h (≈ 1 h 16 min 48 s), as recorded in the training log.

### Model evaluation

The evaluation phase tests model performance through independent picture datasets which were excluded from training to ensure unbiased assessments. The evaluation process matches model-generated bounding boxes with class labels to actual ground truth data for detection and classification accuracy measurements . The evaluation metrics consist of accuracy together with precision recall and mAP. The evaluation process assesses model performance while establishing if further development is needed for practical deployment.

### Detection output generation

The generation of final results following the processing of input photos by the trained model is known as detection output generation. The model identifies landmarks through bounding box creation followed by class label assignment which enables object detection . The detections appear on original X rays through graphical representation which includes color-coded boxes and labels for simplicity. The stage enables simple result verification as well as subsequent research and decision-making applications . The model predictions are presented through an accessible visual representation which provides clear understanding to the users.

### Experimental results

This section presents the experimental results from the proposed model and explains their significance. The results are examined, compared, and discussed to highlight performance, trends, and real-world implications .

**Fig. 3:**
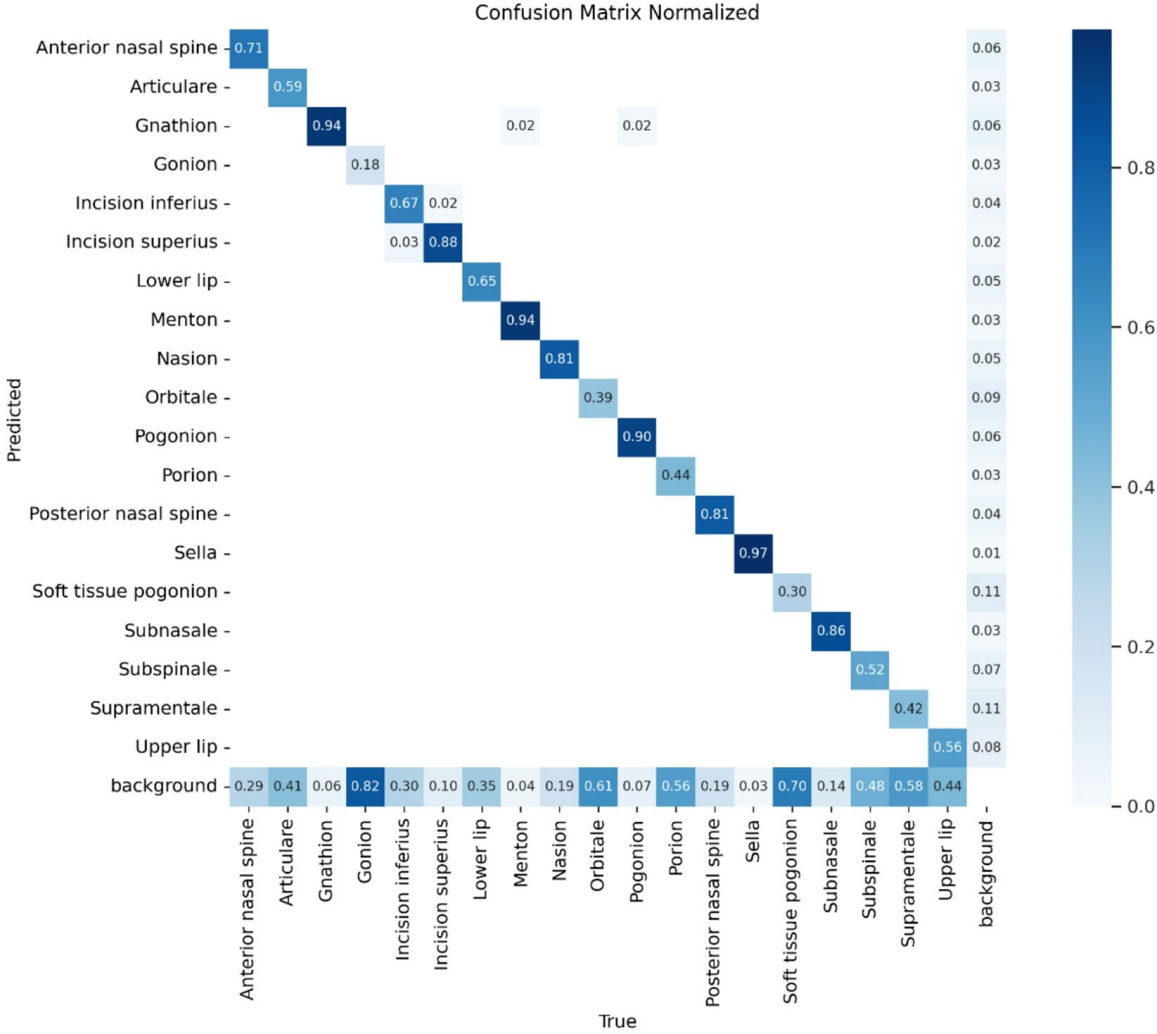
Confusion matrix normalized of the YOLOv12 model’s performance.

The Fig. 3 illustrates the normalized confusion matrix representing the classification performance of the model across multiple cephalometric landmarks. Each cell indicates the proportion of predictions (ranging from 0 to 1) for a given true label (horizontal axis) and predicted label (vertical axis). Higher diagonal values signify accurate landmark localization, while off-diagonal values indicate misclassifications. Notably, landmarks such as Gnathion, Menton, and Sella exhibit high prediction accuracy (≥ 0.9), reflecting the model’s strong capability in identifying these anatomical points. In contrast, moderate confusion is observed in landmarks such as Articulare and Orbitale, suggesting partial overlap or structural similarity in radiographic features.

The test set, comprising 94 images, was used to evaluate the model’s performance. A custom Python script was employed to calculate the error, defined as the distance between the center point of the ground truth bounding box and that of the predicted bounding box.

Table 2 presents the landmark-wise localization accuracy of the model using 1 mm and 2 mm error thresholds. High accuracy is observed for anatomically well-defined landmarks such as Sella, Incision superius, Gnathion, and Menton, with more than 75% of predictions within 1 mm and over 93% within 2 mm. In contrast, landmarks with less distinct boundaries, including Gonion, Subspinale, and Orbitale, show comparatively lower precision, indicating higher localization difficulty. Overall, the model achieves 53.47% accuracy within 1 mm and 80.57% within 2 mm .

**Table 2:** Percentatge of landmarks within 1 mm and 2 mm.

Landmark name	Within 1 mm (%)	Within 2 mm (%)
Anterior nasal spine	32.98	70.21
Articulare	37.23	61.70
Gnathion	76.60	97.87
Gonion	15.96	47.87
Incision inferius	47.87	80.85
Incision superius	82.98	93.62
Lower lip	73.40	97.87
Menton	76.60	96.81
Nasion	58.51	82.98
Orbitale	26.60	62.77
Pogonion	70.21	96.81
Porion	38.30	65.96
Posterior nasal spine	54.26	90.43
Sella	86.17	98.94
Soft tissue pogonion	43.62	76.60
Subnasale	70.21	92.55
Subspinale	19.15	52.13
Supramentale	38.30	75.53
Upper lip	67.02	89.36
Overall	53.47	80.57

**Fig. 4:**
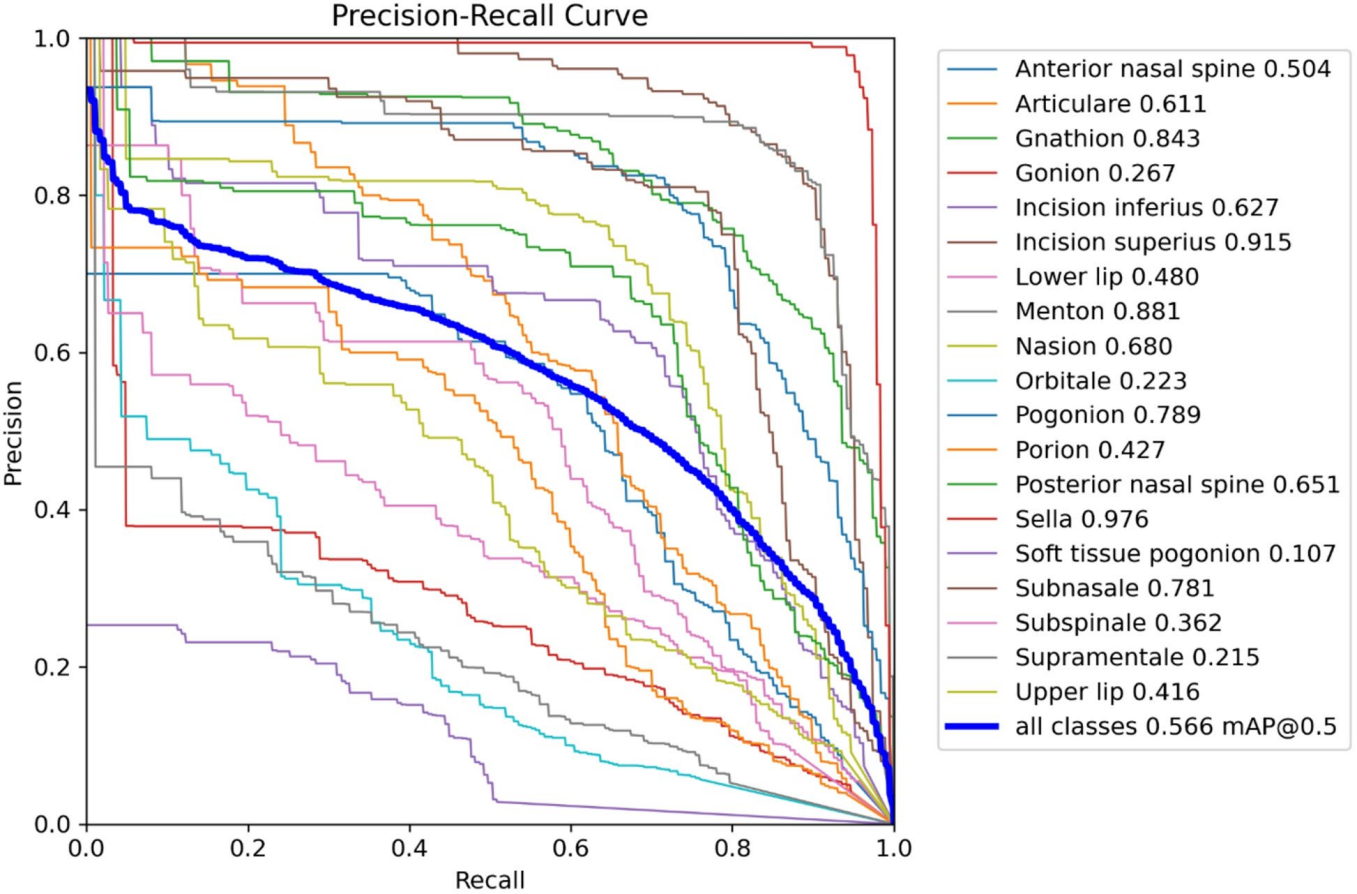
Precision–recall curve for cephalometric landmark detection.

The Fig. 4 presents the precision–recall (PR) curves for all cephalometric landmarks, illustrating the trade-off between precision and recall across varying confidence thresholds. Each colored line corresponds to an individual landmark, while the bold blue curve represents the overall model performance with a mean average precision (mAP@0.5) of 0.566. Landmarks such as Sella (0.976), Incision superius (0.915), and Menton (0.881) achieved high precision–recall values, indicating strong localization and classification accuracy. Conversely, lower performance is observed for landmarks like Soft tissue pogonion (0.107) and Orbitale (0.223), reflecting greater prediction variability in these regions. Overall, the PR curve demonstrates stable model behavior with high precision across most anatomical landmarks, confirming effective generalization and robust feature detection in cephalometric imaging.

**Fig. 5:**
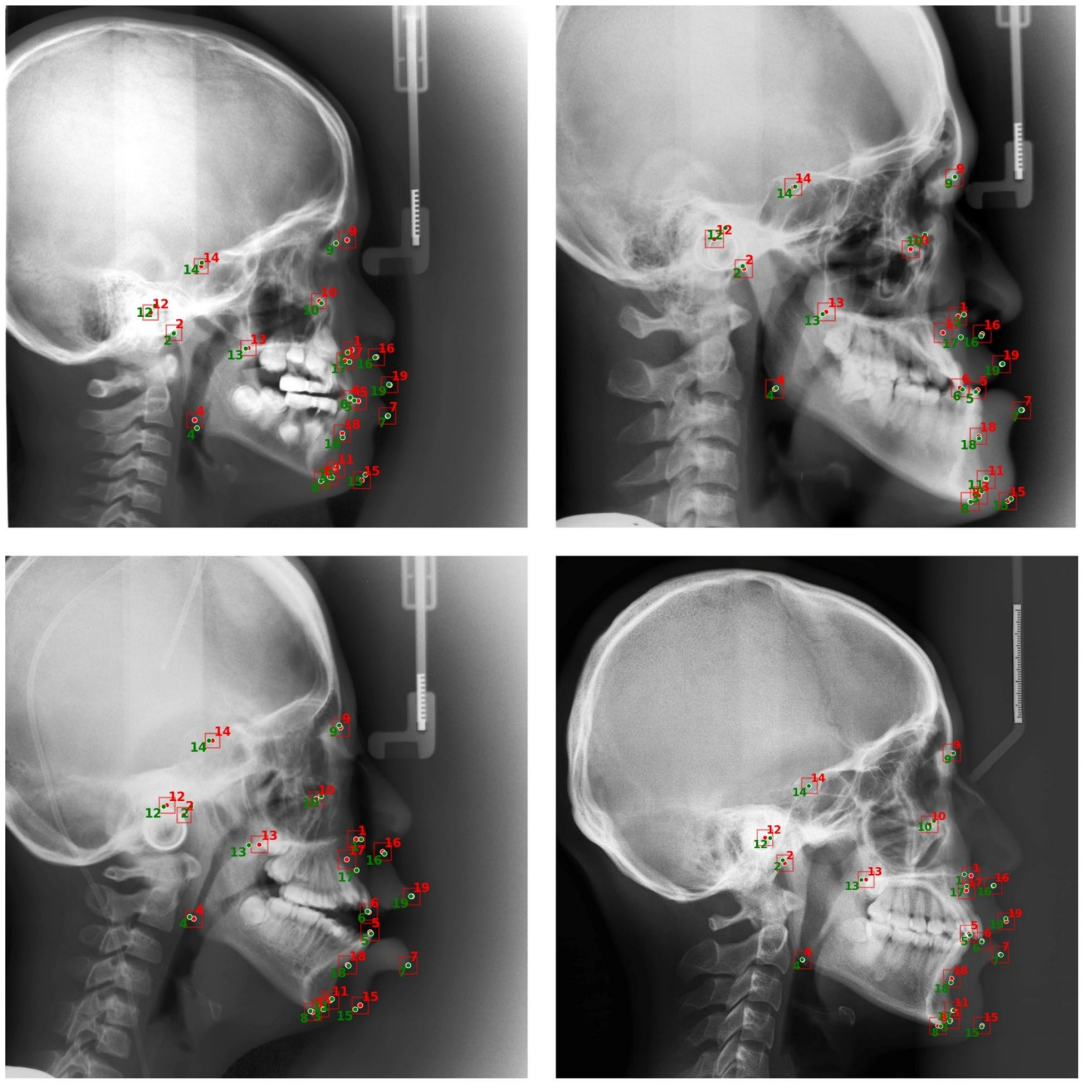
YOLOv12-based cephalometric landmarks predictions and ground truths on varying skull sizes.

The Fig. 5 displays qualitative results of the proposed model on representative cephalometric X-ray images. Each image illustrates automatically detected landmarks in red colour overlaid with their corresponding ground truth annotations in green colour. The close alignment between predicted and actual landmark positions indicates high model precision and consistency in anatomical localization.

**Fig. 6:**
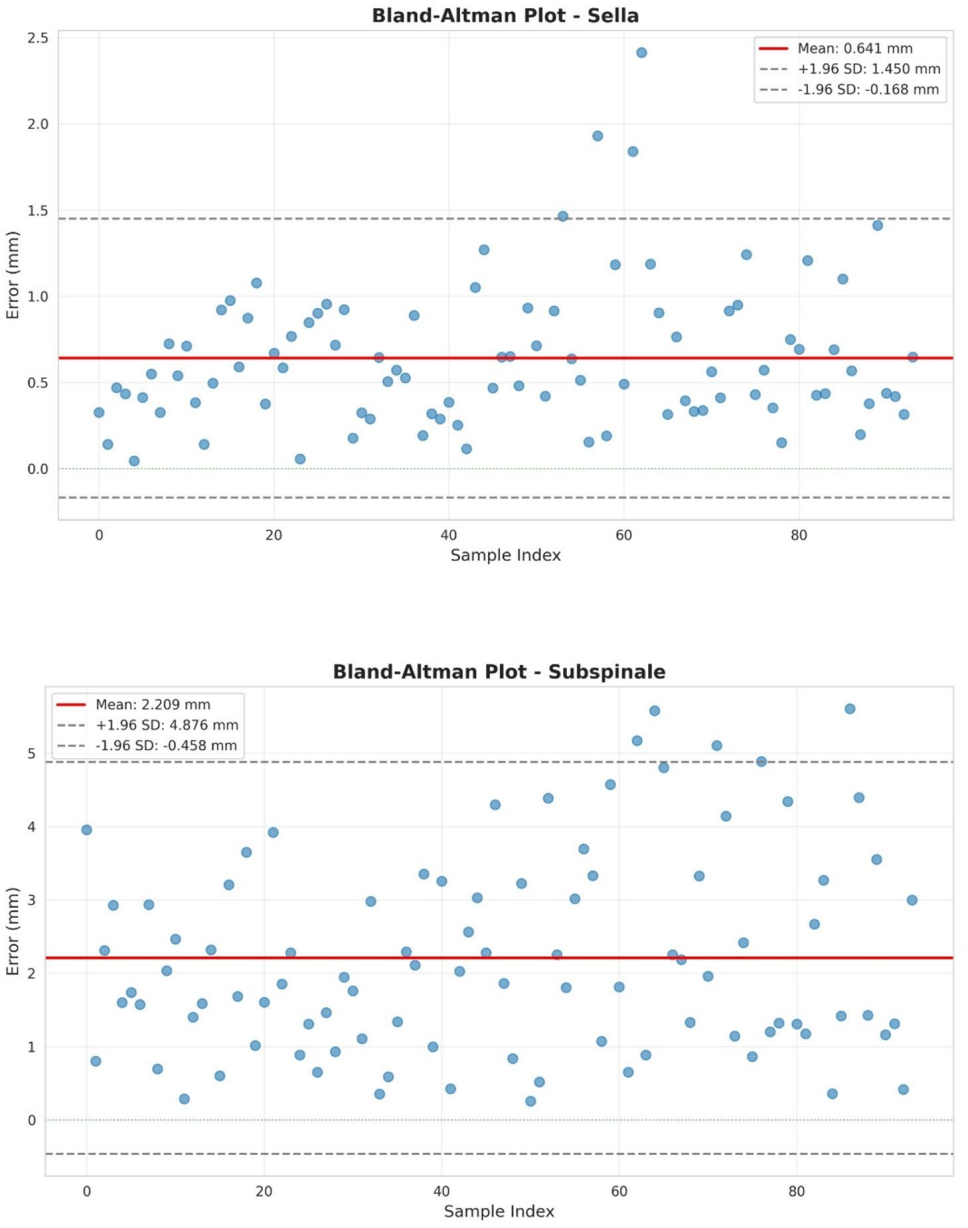
Bland Altman plots for Sella and Subpinale.

The Fig. 6 displays Bland–Altman plots for the Sella and Subspinale landmarks. For Sella, the mean bias is 0.64 mm with narrow limits of agreement (− 0.17 to 1.45 mm), and most errors cluster close to the mean, indicating good agreement. On the other hand, Subspinale exhibits a larger mean bias of 2.21 mm and considerably wider limits (− 0.46 to 4.88 mm), with several observations near the upper bound, reflecting greater variability and occasional large deviations. Together, these plots indicate robust and consistent localization for Sella while highlighting the need for model refinement for Subspinale.

## Discussion

The performance analysis of the model reveals several important insights into its landmark detection capabilities. The localization accuracy varied among the landmarks. Notably, the Articulare and Porion landmarks demonstrated higher error values, indicating that these points require further improvement in detection accuracy. The elevated localization errors can be attributed to the superimposition of bilateral structures in lateral cephalometric projections, which creates overlapping anatomical contours that obscure precise identification. Additionally, both landmarks reside in regions of inherently low radiographic contrast, thereby reducing feature discriminability and compromising YOLOv12’s detection performance in these anatomically complex areas.

In contrast, the model performed well with closely positioned landmarks such as Gnathion, Menton, and Pogonion, without showing signs of confusion between them. Similarly, it was able to correctly distinguish between Incision Inferius and Incision Superius, which are anatomically close but visually distinct points. This suggests that the model has learned spatial relationships and visual cues beyond simple pixel-wise matching.

Landmarks such as Sella and Nasion were consistently detected with high accuracy, reflecting the model’s strong ability to identify points with well-defined anatomical features.

However, other landmarks exhibited moderate errors, suggesting some variability in performance depending on the landmark’s location, visibility, or surrounding anatomical structures. Importantly, the model showed robust performance across varying skull sizes, which indicates that it has learned generalizable patterns rather than merely memorizing the training data.

One of the notable limitations observed is that certain landmarks, particularly those located around the chin region including Gnathion, Menton, Pogonion, and Soft Tissue Pogonion tend to be affected by X-ray image quality. If the X-ray contains white patches, is overexposed at the bottom, or appears “flushed,” the model’s accuracy in detecting these landmarks decreases significantly.

Additionally, during training, some landmarks exhibited low confidence scores, which may impact their detection consistency. This points to the importance of both high-quality annotations and balanced training data to improve model confidence and reliability.

YOLO, a bounding box-based detector, was adapted for landmark detection by using the box center as the landmark. While this adaptation leverages YOLO’s efficiency and robustness, it may introduce minor precision loss compared to heatmap regression methods, which explicitly predict continuous-valued landmark positions and are traditionally favored for pinpoint localization tasks. The fixed box size of 58 × 58 pixels, chosen based on the resolution of cephalometric images, ensures that the anatomical region of interest is adequately captured without introducing ambiguity. With a spatial resolution of 0.1 px/mm, this configuration provides sufficient granularity to localize the landmark within moderate tolerances. This balance between computational efficiency and localization accuracy makes YOLO a viable option for landmark detection, especially in high-throughput or resource-constrained environments.

The use of data augmentation during training contributed to improved robustness and generalization, helping the model handle variability in image quality and patient anatomy. However, the quality of input X-rays remains a critical factor affecting performance.

## Conclusion

In this study, a deep learning-based model was developed to automatically detect 19 cephalometric landmarks on lateral X-ray images. While certain points like Sella, Nasion, Menton, and Pogonion were detected with high precision even when closely positioned the performance on landmarks such as Articulare and Porion indicates room for improvement. The model demonstrated strong generalization across varying skull sizes, suggesting effective learning rather than memorization. However, its performance was sensitive to the quality of X-ray images, especially in overexposed or low-contrast regions. Although not yet suitable for clinical deployment, the model is a proof of concept where it can as an assistive tool in cephalometric analysis, where predictions can be refined by users within a semi-automated workflow. With further improvements in data quality, model training, and integration into user-interactive systems, this work lays the foundation for more robust and practical AI-assisted cephalometric tools.

### Future scope

While the model does not yet achieve clinical-grade accuracy, it serves as a valuable proof of concept. It shows potential as an assistive tool in cephalometric analysis, where predicted landmarks can be manually adjusted by users in specialized software for calculating cephalometric angles. Such an approach would combine the speed of automation with the precision of expert correction. The model needs improvement in the Cephalometric landmarks with low confidence scores. Training on a larger and more diverse dataset, representing a wider range of skull anatomies, ethnicities, and age groups, would enhance the model’s generalization. Inclusion of clinical cases with abnormalities could also test the robustness of the model in real-world scenarios. With continuous improvement in model architecture, data quality, and training techniques, future versions of this system may move closer to clinical-grade accuracy, enabling deployment in diagnostic, educational, and treatment planning environments.

## Data Availability

The dataset used in this study is openly available online at Roboflow: https://universe.roboflow.com/cephalometric-hbcxy/cephalometric-6hhqy
